# Predictive brain signals mediate association between shared reading and expressive vocabulary in infants

**DOI:** 10.1371/journal.pone.0272438

**Published:** 2022-08-03

**Authors:** Shinmin Wang, Ovid J. L. Tzeng, Richard N. Aslin

**Affiliations:** 1 Department of Human Development and Family Studies, National Taiwan Normal University, Taipei, Taiwan; 2 Department of Educational Psychology and Counseling, National Taiwan Normal University, Taipei, Taiwan; 3 Taipei Medical University, Taipei, Taiwan; 4 Linguistic Institute, Academia Sinica, Taipei, Taiwan; 5 Haskins Laboratories, New Haven, CT, United States of America; 6 Department of Psychology and Child Study Center, Yale University, New Haven, CT, United States of America; 7 Department of Psychological Sciences, University of Connecticut, Storrs, CT, United States of America; The University of Edinburgh, UNITED KINGDOM

## Abstract

The ability to predict upcoming information is crucial for efficient language processing and enables more rapid language learning. The present study explored how shared reading experience influenced predictive brain signals and expressive vocabulary of 12-month-old infants. The predictive brain signals were measured by fNIRS responses in the occipital lobe with an unexpected visual-omission task. The amount of shared reading experience was correlated with the strength of this predictive brain signal and with infants’ expressive vocabulary. Importantly, the predictive brain signal explained unique variance of expressive vocabulary beyond shared reading experience and maternal education. A further mediation analysis showed that the effect of shared reading experience on expressive vocabulary was explained by the infants’ predictive brain signal. This is the first evidence indicating that richer shared reading experience strengthens predictive signals in the infant brain and in turn facilitates expressive vocabulary acquisition.

## Introduction

Human language development involves a complex interplay of neurobiological and environmental factors [e.g., 1, 2]. Recently, at the neurobiological level, the neural response associated with top-down sensory prediction (i.e., the ability to predict future events based on prior sensory information) was suggested as one mechanism that could facilitate infants’ expressive (spoken) vocabulary development [3]. However, it is unclear whether the relation between predictive brain signals and expressive vocabulary is due to a maturational process or to the effects of early experience. Among the environmental factors, the role of early shared reading experience in language development during early childhood has been widely recognized [e.g., 4]. The American Academy of Pediatrics (AAP) thus recommends that parents read to their children soon after their birth [5]. And, there are many early interventions involving shared reading [see 6 for a review], such as dialogic reading (a specific reading technique that encourages parents to involve the child actively in verbal interactions during shared book reading) [7] and Reach Out and Read [8, 9]. What remains untested is how the interplay of early shared reading experience and the brain’s predictive signals influences language development. In the current study, we explored the role of early shared reading experience in the formation of predictive brain signals and then asked whether predictive brain signals account, at least in part, for the observed relation between early shared reading experience and expressive vocabulary development during infancy.

### Shared book reading in early childhood

Parent-child shared book reading has long been promoted to boost children’s language and cognitive development even in infancy [5]. Close links have been established between shared book reading experience in early childhood and subsequent expressive vocabulary development [e.g., 4, 10, 11]. Mol et al. [4] indicated that dialogic parent-child book reading explains 8% of the variance in expressive vocabulary of 2- to 6-year-old children in their meta-analysis, with the effect being even larger when children were younger (2 to 3 years old). Very similar explanatory power (about 8%) was also noted among preschoolers in an earlier meta-analysis conducted by Bus et al. [12], suggesting the effect of reading to children on children’s linguistic competence is fairly reliable across studies [for counter evidence see e.g., 13]. Although this amount of explained variance is not very high, reading to children can be more easily manipulated than, for example, the socioeconomic status of the family [14]. And the initial advantages created by a positive home literacy environment would accumulate over time [e.g., 11, 14–16].

Importantly, recent evidence suggests that parent-child shared book reading may impact language processing in young children’s brain [17–19]. Greater home reading exposure was positively associated with activation of brain areas supporting semantic language processing (i.e., the left-hemisphere parietal-temporal-occipital cortex) among nineteen 3- to 5-year-old children while listening to stories [17]. A follow-up study of 4-year-olds further identified that the influence of shared book reading on brain activations was not restricted to classic language processing areas [18]. While listening to stories, children with mothers who read to them more interactively had greater brain activations not only in the brain regions supporting language processing (i.e., left inferior frontal gyrus) but also in the areas associated with executive function (i.e., frontal pole, temporal pole).

Executive function refers to a higher level attentional control system that is engaged in many tasks, including language processing, and has been found to play a key role in language development in early childhood [e.g., 20]. This suggests that the benefit of shared reading experience to brain development may also manifest itself in other brain functions that have been identified to be important in language processing, such as top-down sensory prediction [3]. In the current study, we therefore investigated the link between infants’ shared reading experience and their neural response in a top-down sensory prediction task, a task previously identified to be related to infants’ expressive vocabulary development [3].

### Predictive brain of infants

Top-down sensory prediction is one of the extraordinary capacities already present in young infants that may facilitate, and even be necessary for, efficient transmission of information in the brain [21–23]. Predictive signals in the infant brain have been revealed as early as 6 months of age using fNIRS [24]. In Emberson et al., 6-month-old infants were exposed to two novel audio-visual pairs followed by visual omission trials, where the predicted visual stimulus is unexpectedly omitted. Results showed that a robust occipital response was present on visual-omission trials, but only after the auditory cue had been reliably paired with the visual stimulus.

A follow-up study [34] showed that the occipital responses on visual-omission trials were absent in prematurely born infants tested at 6 months of (corrected) age, a population at high risk of subsequent language delay [e.g., 25]. Recently, these predictive fNIRS signals measured at 6 months of age were found to longitudinally relate to full-term infants’ expressive vocabulary at 12 months and 18 months of age [3]. This observed longitudinal link between the brain’s predictive signal and vocabulary development is consistent with the hypothesis that predictive processing fundamentally shapes language processing in the human brain [26].

### Shared book reading and predictive brain

How might parent-infant shared reading experience play a role in the formation of robust predictive brain signals? We know that adults tend to spontaneously interact with their infants using a range of strategies such as prediction and inference during shared book reading [e.g., 27, 28], even when their infants still possess rudimentary spoken language skills [29]. For example, mothers of 14-month-old infants start to coax their infant to predict forthcoming events based on the story they are reading [27]. Reading regularly to infants and young children may thus provide them with opportunities to engage in predicting forthcoming information in a relatively well-structured and well-informed context. This may in turn provide them with practice in forming predictive abilities at the neural level. If this is the case, a positive correlation between shared reading experience and the strength of the brain’s predictive signals would be expected.

If a positive correlation between shared reading experience and the strength of the brain’s predictive signals is found, a further interesting question is whether the predictive brain signals account, at least in part, for the well-established relation between early shared reading experience and expressive vocabulary development. This question is motivated by previous research that investigated how the interplay of environmental input and brain function influences children’s language development and has revealed the brain function as a mediator of the link between environmental input and language development. For example, Romeo et al. [30] found that the number of adult-child conversational turns was positively linked with brain activation in left inferior frontal regions (Broca’s area) during story listening among 4-to-6-year-old children. And this neural measure significantly mediated the relation between conversational turns and children’s language skills.

### The present study

In the current study, we follow a similar line of reasoning to test a model in which the brain’s predictive signals serve as a mediator of the link between early shared reading experience and infants’ expressive vocabulary. To achieve these aims, we tested 12-month-old infants’ predictive brain signals using functional near-infrared spectroscopy (fNIRS), their parent-child shared reading experience as assessed by the StimQ-READ subscale-Infant [31, 32] translated into Chinese, and their expressive vocabulary as assessed by the Infant form of the Mandarin-Chinese Communicative Development Inventory (Taiwan) (hereafter MCDI-T) [33]. We hypothesized that the parent-child shared reading experience would be positively correlated with the strength of the predictive brain signal and that the predictive brain signal would serve as a mediator that explains the underlying mechanism of the relation between parent-child shared reading experience and infants’ expressive vocabulary development.

## Materials and methods

### Participants

The sample size of this study was determined based on the effect size (*d* = 0.61) reported in previous research that is required to detect the predictive brain signals [34]. Using G*Power, for a significance level of .05 (one-tailed) and power of .8, the adequate sample size to detect the occipital lobe response to the unexpected omission of a visual stimulus in full-term infants was 19. After also taking into account the typical exclusion rate (40–60%) as reported in fNIRS infant studies [e.g., 35–37], the present study initially recruited a total of 51 healthy infants aged 12 months (projecting the 63% exclusion rate).

Among the 51 infants, 28 infants (54%) were excluded from the analysis for the following reasons: refusal to wear the fNIRS caps (*n* = 3), cried before starting data collection (*n* = 4), excessive movement (*n* = 6), interference by caregiver (*n* = 3), failure to watch a sufficient number of trials during the familiarization phase (*n* = 4) or during the test phase (*n* = 8) (see detailed exclusion criteria below). Thus, the final sample was 23 infants (gender: 16 males; chronological age: *M* = 12.48 months, *SD* = 0.95 months; gestational age at birth: *M* = 37.83 weeks, *SD* = 1.67 weeks, range = 34–40 weeks).

The final sample of 23 infants and their parents were all native Mandarin Chinese. All infants had no known hearing or vision impairment. At the time of testing, 19 infants (83%) were the only child in their families, and 16 infants (70%) came from nuclear families (i.e., a couple and their dependent children). Primary caregivers during daytime include mother (13 infants, 56.5%), grandmother (3 infants, 13.0%) and nanny (4 infants, 17.4%) (3 infants have missing data). Primary caregivers at night are mothers (18 infants, 78.3%), father (4 infants, 17.4%) and grandfather (1 infant, 4.3%). Most mothers had a college degree (n = 14, 60.9%), with the remaining (n = 9, 39.1%) having a postgraduate degree as their highest level of education. Most fathers had a college degree (n = 12, 52.2%). 9 fathers had a postgraduate degree (39.1%) and the remaining (n = 2, 8.7%) had a 5-year college degree. Participants were recruited through online advertisement in 2020 and the database of interested participants for the NTNU-Haskins Joint Laboratory of Brain Development and Learning. This study was approved by the Institutional Review Board of National Taiwan Normal University and informed parental consent was obtained prior to participation. After consenting, parents (father or mother) completed the measure of expressive vocabulary (MCDI-T) and the measure of early shared reading experience (StimQ-READ) before or after the fNIRS task, depending on the readiness of the infant.

### Measures

#### Measure of expressive vocabulary: MCDI-T

The expressive vocabulary of the infants was assessed using the Word part of the Infant form of the Mandarin-Chinese Communicative Development Inventory (Taiwan) (hereafter MCDI-T) [33], a parental report widely used in Taiwanese studies [e.g., 38, 39]. Parents were shown a checklist of 354 vocabulary items and asked whether their child uses each word in their expressive vocabulary. Number of words produced was calculated and converted to percentile scores based on the Taiwanese norms established for boys and girls, respectively.

#### Measure of shared reading experience: StimQ-READ

Infants’ early shared reading experience was measured via a 10-minute structured interview during the lab visit using the Infant version of StimQ-READ subscale [31, 32], an orally administrated questionnaire measuring cognitive stimulation in the home environment. The scale was translated into Chinese by the first author. It consists of questions regarding reading frequency, book content and verbal labeling. Three questions related to the book reading frequency included: number of books and board books, and days of book reading per week. Eight questions related to the book content are: whether parents read books containing nursery rhyme, daily activities, body parts, shapes, things around the house, toys and child’s favorite things, animals, and photographs of babies. One question concerns whether parents label pictures while reading to their child. The maximum score is 19 (see S1 Appendix in S1 File for scoring). The StimQ was validated in past studies [31, 32] by showing that it was significantly correlated with (*r* = .55) the Home Observation for Measurement of the Environment (HOME), the gold standard observational measure of the home environment. It has been successfully used to investigate how reading exposures related to brain areas supporting language processing in English-speaking children [17, 19] and to index the amount of reading exposures in Mandarin-Chinese populations [29].

#### Measure of predictive brain signals: fNIRS

*fNIRS task*. The fNIRS task was presented using E-prime 2. The stimuli, two pairs of audio-visual stimuli (A1V1, A2V2), are the same as those used in Emberson, Richards, and Aslin [24] and can be downloaded from that study. The stimulus presentation procedure was also adapted from Emberson et al. [24], consisting of repeatedly paired auditory-visual events followed by rare auditory-only events (visual-omission trials) (see S1 fNIRS in S1 File task for detailed descriptions about stimuli and the stimulus presentation procedure).

**Fig 1:**
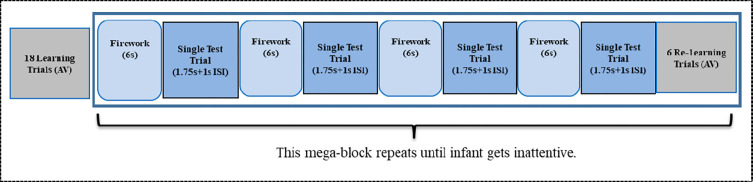
The fNIRS task procedure.

The task included a familiarization phase and a test phase. In the familiarization phase, 18 trials (nine A1V1 and nine A2V2) were presented in random order and separated by a interstimulus interval (ISI) for 1s. After the familiarization phase lasting 49.5 seconds (1750ms*18 trials + 1000ms*18 ISIs), the test phase consisted of at most 8 mega-blocks. Each mega-block consisted of four test trials followed by a mini re-learning block (Fig 1). The four test trials included two ‘*standard audiovisual test trial*’ (A1V1 or A2V2) and two ‘*visual-omission test trial*’ (A1V- or A2V-) presented randomly. Each test trial was followed by a 1s ISI and then 6s dimmed firework video. This design ensures only 20% of the trials consisted of unexpected visual omissions, thereby maintaining sensory expectations for A1V1 and A2V2 pairings over the duration of the experiment. The mini re-learning block was composed of 6 trials (three A1V1 and three A2V2) presented randomly with a 1s ISI between each trial. These re-learning trials were designed to ensure that the learning of AV pairings was maintained after a series of test trials. Each infant viewed a different number of mega-blocks, depending on how long they maintained interest in the displays.

**Fig 2:**
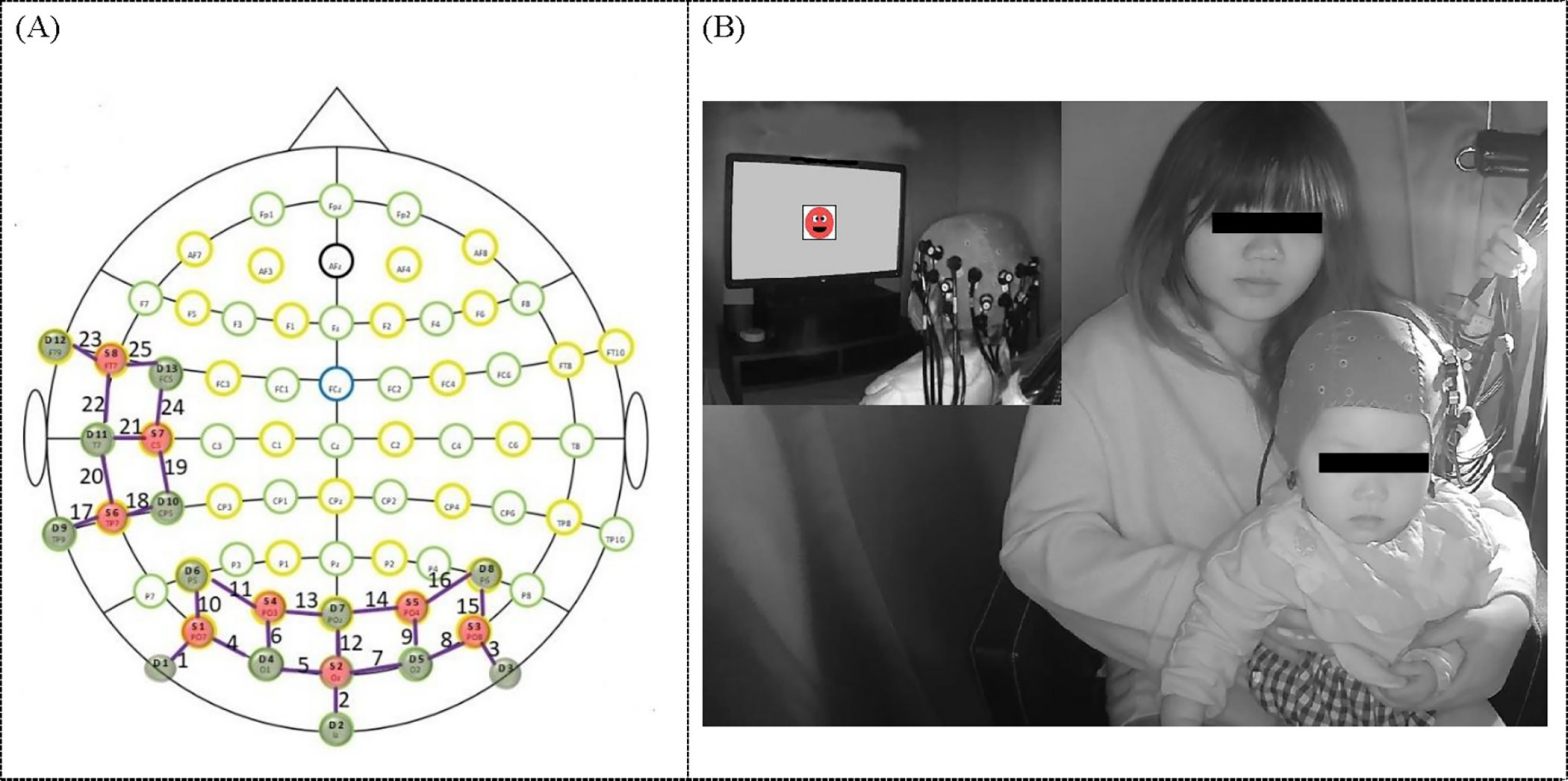
(A). Channel placement over occipital and temporal areas. Red circles indicate sources and green circles indicate detectors. (B). Picture of a 12-month-old sitting in his mother’s lap inside the blackout-curtain-surrounded area during the experiment. The panel inserted in the upper left corner shows the back view of the infant with cap placed upon the head, and the schematic depicts the screen from the infant’s point of view.

*fNIRS recording*. The fNIRS data were collected using a NirScout (NIRx Medizintechnik GmbH, Berlin, Germany) CW-NIRS device with 25 channels (8 light emitters, λ1∣2 = 760∣850 nm with a power of 5 mw/wavelength, and 13 light detectors) sampled at 7.81 Hz. Data were converted to concentration changes using the modified Beer-Lambert law (mBLL). The distance between light emitters and detectors was 2.5 cm. With reference to the EEG 10–20 system, the 8 light emitters and 13 light detectors were arranged in two grids covering bilateral occipital cortex (16 channels) and left middle temporal gyrus (9 channels) (Fig 2A, 2B). For the primary purpose of the current study, only data from channels over the occipital cortex were reported here.

Before the experiment, the size of the infant’s head was measured and the best-fitting EasyCap (44, 46, 48, 50 cm) was chosen before the experiment. Mean head size of the 23 infants was 45.84 cm (*SD* = 1.45, *Min*. = 43, *Max*. = 48). To position the cap, we measured the distance from the Nasion to the Inion centrally over the head of each infant with a flexible tape, put on the cap and placed the standard 10–20 position Cz half-way and made sure the cap was left-right symmetric (used the middle point between the bilateral preauricular points as a reference).

During the experiment, infants sat on their parent’s lap approximately 60 cm from a 23-inch computer monitor in a blackout-curtain-surrounded area in a lab room (Fig 2B). A camera above the screen recorded infants’ behavior to allow offline coding of attention and movement throughout the experiment.

*fNIRS data analysis*. Infants’ attention to visual stimuli shown on screen and their movements during the fNIRS recordings were coded offline from the experimental videos. Infants who viewed fewer than 12 out of 18 trials during the familiarization phase, or who viewed fewer than 4 single trials on either type of test trial (AV, AV-) were excluded from further analyses (see *Participants* section for the number of infants excluded).

The final sample of 23 infants contributed an average of 16.91 AV learning trials during the familiarization phase (*SD* = 1.44, *range* = 14–18) and watched an average of 3.83 mega-blocks (*SD* = 1.15, *range* = 2–6) during the test phase. The mean total experimental duration was 4.11 minutes (*SD* = 0.99, *range* = 2.54–5.98). After discarding trials that infants did not pay attention to, infants looked at an average of 7.87 AV trials (*SD* = 2.22, *range* = 4–12) and of 7.57 AV- trials (*SD* = 2.35, *range* = 4–12) during test.

The fNIRS data were processed using the Homer 2 package in MATLAB R2018b [40], following the processing stream recommended by Di Lorenzo et al. [41] for infant data (see S1 fNIRS data processing in S1 File). Then channels showing significant changes during both types of single test trials (AV, AV-) were identified as our channels of interest (channel 15 & 16) (see S1 identification of channels of interest in S1 File). Channel 15 and channel 16 approximately correspond to placement of PO4/P6/PO8 (see Fig 2A). Oxy-Hb and deoxy-Hb concentration changes of channel 15 and channel 16 were then averaged over participants and two types of single test trials (AV, AV-) and used for subsequent analyses.

## Results

Descriptive statistics for the principal measures and how they were related to each other are summarized in Table 1.

**Table 1:** Correlation coefficients (*r*) between measures and descriptive statistics.

	1	2	3	4	5	6	7	8
1. Gestational age (week)	1							
2. Birth weight (gram)	.591**	1						
3. Shared reading experience (raw)	-.024	-.034	1					
4. Words produced (PR)	-.078	-.038	.494**	1				
5. Occipital_AV (oxy-Hb)	-.064	.263	-.248	-.303	1			
6. Occipital_AV- (oxy-Hb)	-.238	.160	.440*	.599**	.011	1		
7. Occipital_AV (deoxy-Hb)	-.227	-.122	-.303	-.391	.205	-.316	1	
8. Occipital_AV- (deoxy-Hb)	-.446*	-.111	-.053	-.299	.029	-.351	.396	1
*Mean*	37.826	2992.430	12.261	47.565	.011	.015	-.004	-.009
*SD*	1.669	618.923	3.320	22.653	.011	.015	.008	.008
*Min*.	34	1698	7	15	-0.005	-0.011	-0.023	-0.027
*Max*.	40	4690	19	99	0.031	0.042	0.013	0.002

** *p*< .01 (1-tailed)

*p< .05 (1-tailed); Pairwise N = 18~23 (item1~4, N = 23; item5~8, N = 18).

### Predictive brain signals on visual-omission trials

**Fig 3:**
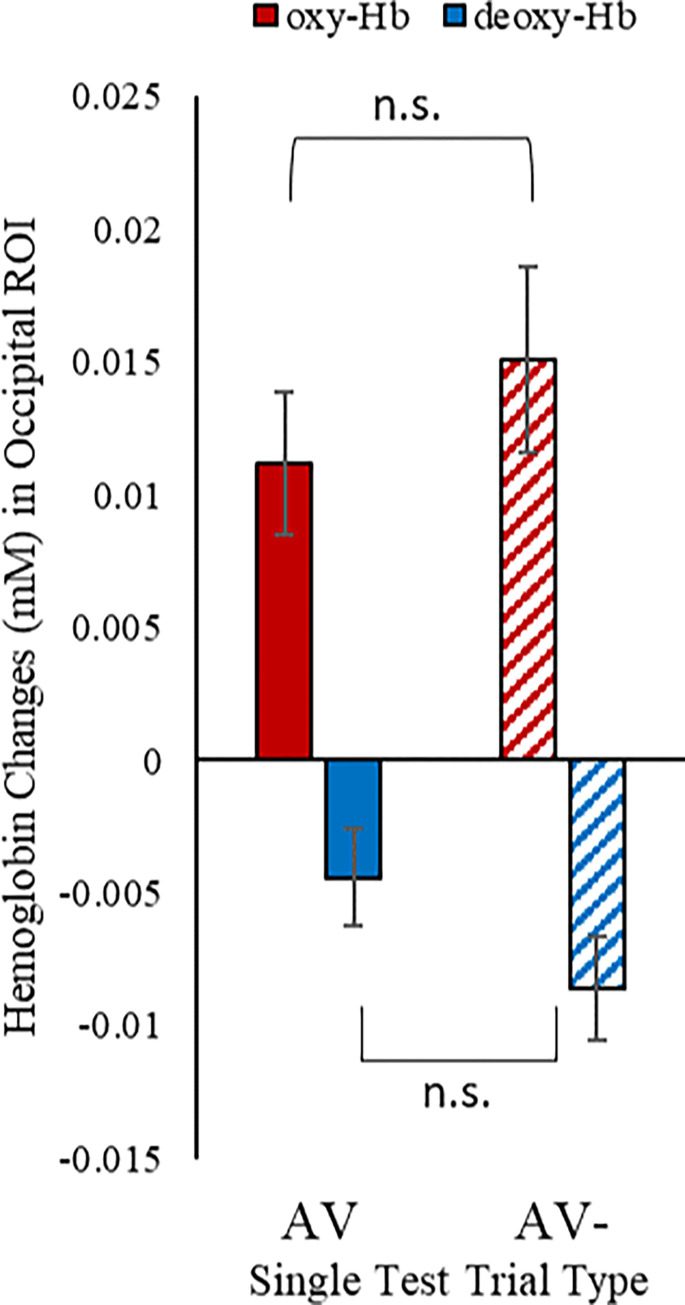
Group-averaged bar-plots (with standard errors as error bars) in oxy-Hb and deoxy-Hb signals in response to standard audiovisual (AV) test trials and visual-omission (AV-) test trials respectively.

Fig 3 displays the group-averaged bar-plots in oxy-Hb and deoxy-Hb signals in occipital ROI in response to the AV test trials and AV- test trials. Statistical analyses showed significant positive changes in oxy-Hb [mean ± SD: 0.011 ± 0.011 mM (95% CI: 0.006, 0.017); one sample t-test (two-tailed, against zero baseline), *t*(17) = 4.17, *p* = .001, Cohen’s *d* = 1.0] and a significant decrease in deoxy-Hb [mean ± SD: -0.004 ± 0.008 mM (95% CI: -0.008, -0.001); one sample t-test (two-tailed, against zero baseline), *t*(17) = -2.41, *p* = .028, Cohen’s *d* = 0.5] on the AV test trials.

Critically, one sample t-test (two-tailed) against zero baseline also revealed significant positive changes in oxy-Hb [mean ± SD: 0.015 ± 0.015 mM (95% CI: 0.008, 0.023); *t*(17) = 4.31, *p* < .001, Cohen’s *d* = 1.0] and a significant decrease in dexoy-Hb [mean ± SD: -0.009 ± 0.008 mM (95% CI: -0.013, -0.005); *t*(17) = -4.37, *p* < .001, Cohen’s *d* = 1.1] in response to the AV- test trials. Thus, the 12-month-old infants in the present study also showed a robust occipital response to visual-omission trials, in line with findings from the previous work by Emberson et al. [24] in 6-month-old infants.

### Mediation analysis of the predictive brain signal

**Fig 4:**
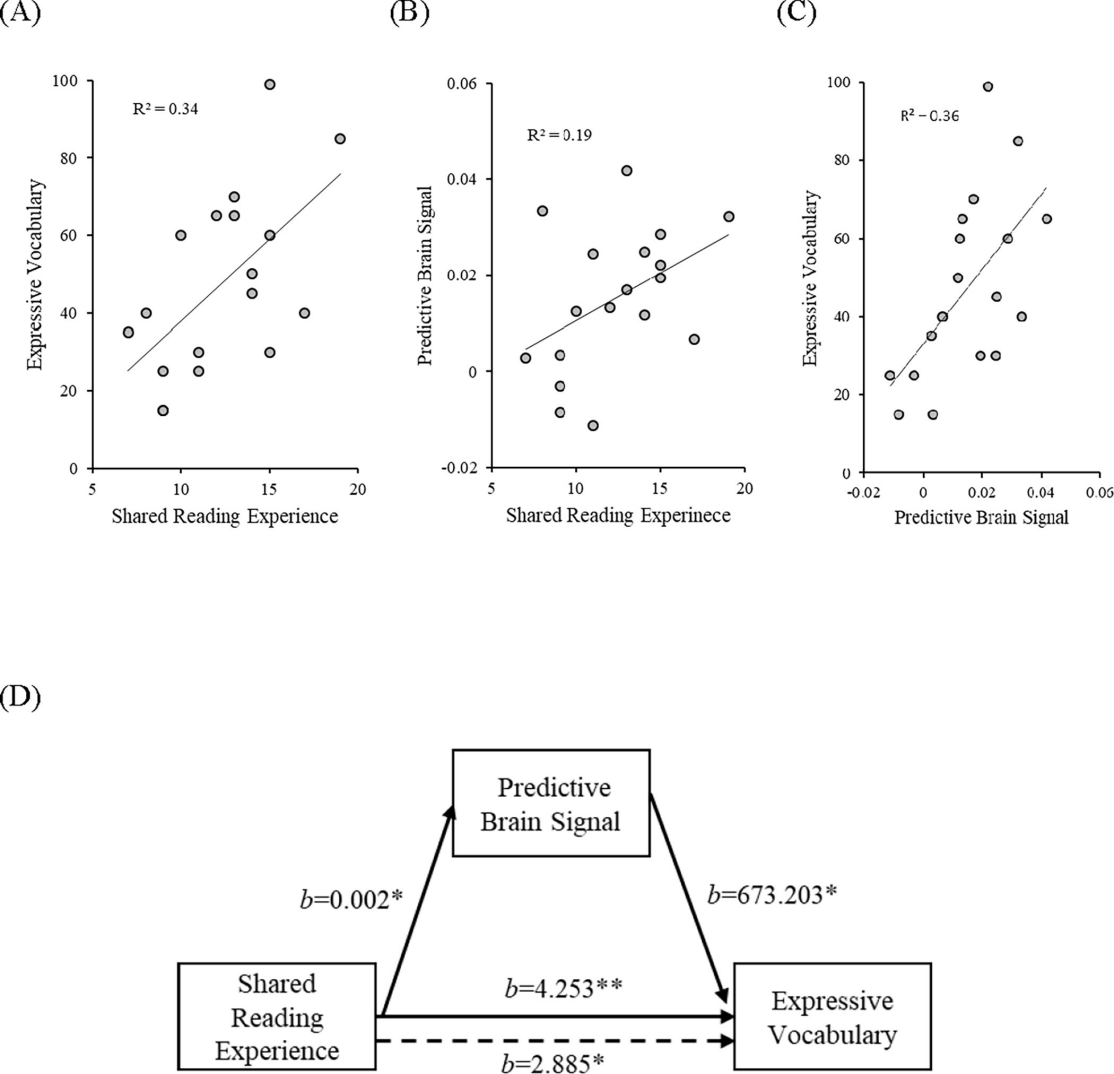
The three scatter plots (with best-fitting regression lines) show zero-order correlations between (A) shared reading experience (raw scores) and expressive vocabulary (PR), (B) shared reading experience (raw scores) and predictive brain signal (oxy-Hb; mM), (C) predictive brain signal (oxy-Hb; mM) and expressive vocabulary (PR). The mediation model (D) shows the effect of shared reading experience on expressive vocabulary, as mediated by predictive brain signal. Along the lower path, the solid and dashed arrows show results when the mediator was not included and was included in the model, respectively. Asterisks indicate significant paths (**p < .01, *p < .05).

The mediation analysis was conducted following the four steps suggested by Baron and Kenny [42]. First, infants’ early shared reading experience (the StimQ-READ total score) was significantly correlated with their expressive vocabulary (*r* = .494, *p* = .008) (also see Fig 4A). Second, infants’ early shared reading experience was also significantly related to the predictive brain signals as indexed by oxy-Hb changes to the visual-omission trials (*r* = .440, *p* = .034) (also see Fig 4B). Then, the predictive brain signals as indexed by oxy-Hb changes to the visual-omission trials was significantly correlated with infants’ expressive vocabulary (*r* = .599, *p* = .004) (also see Fig 4C). This relation remains significant (*r* = .488, *p* = .023) even when infants’ early reading experience was controlled. Further hierarchical regression analysis (see Table 2) revealed that the predictive brain signal (oxy-Hb) was a significant predictor of expressive vocabulary above and beyond maternal education and early shared reading experience and accounted for 13.6% of unique variance in expressive vocabulary. The socioeconomic status of the family (SES) was indexed by maternal education as it has been shown that it was the best predictor of children’s development among other SES indexes such as father education or family income [43, 44].

**Table 2:** Hierarchical regression analysis.

Step	Predictors	Dependent Variable					
		Expressive vocabulary					
		final β	*p*	90% CI	Total *R*^2^	Δ*R*^2^	Δ*p*
1	Maternal education	-.001	.497	〔-16.000, 15.871〕	.003	.003	.409
2	Shared reading experience	.401	.042	〔0.155, 5.615〕	.352	.348	.006
3	Occipital_AV- (oxy-Hb)	.422	.037	〔58.399, 1288.007〕	.488	.136	.037

Listwise, N = 18

Finally, to test whether the predictive brain signal plays a causal role between early shared reading experience and expressive vocabulary (while controlling for maternal education), the simple mediation model was conducted using the PROCESS version 3.5 for SPSS 23 [45, 46]. The indirect effects in the mediation models were tested using 5000 bootstraps samples with 90% confidence intervals. One-sided tests (90% confidence intervals) were used in subsequent analyses as our hypotheses have been formulated with direction (+) [47]. This mediation analysis showed that the effects of early shared reading experience on expressive vocabulary was significant (*b* = 4.253, *p* = .001, CI = [1.6269, 6.8792]) (Fig 4A and 4D). The effect of exposure to early shared reading on brain predictive signal was significant (*b* = 0.002, *p* = .032, CI = [0.0002, 0.0038]; standardized *b* = .45, suggesting a halfway between the values for medium and large effects) (Fig 4B and 4D). The effect of brain predictive signal on expressive vocabulary was also significant (*b* = 637.203, *p* = .037, CI = [58.4109, 1287.9950]; standardized *b* = .42, suggesting a halfway between the values for medium and large effects) (Fig 4C and 4D). Critically, the parameter estimates for the effect of early shared reading experience on expressive vocabulary was reduced from 4.253 to 2.885, as shown in Fig 4D. The magnitude of the predictive brain signals significantly mediated the relation between early shared reading experience and expressive vocabulary (indirect effect = 1.368, 90% CI = [0.0975, 3.2989], indirect/total effect = 0.32), indicating that this neural pattern explained 32% of the relation between early shared reading experience and infants’ expressive vocabulary. This result suggests that early shared reading experience may support infants’ expressive vocabulary in part by influencing infants’ prediction ability at the neural level. The final model explained 59.3% of the variance in infants’ expressive vocabulary abilities.

## Discussion

Although the importance of shared book reading in early language development has been thoroughly documented [e.g., 4, 16], the current study provides the first evidence that demonstrates the neural mechanism underlying the relation between parent-child shared book reading and children’s language development. Using fNIRS, we identified the predictive brain signal (indexed by oxy-Hb changes to the visual-omission trials) as a robust predictor of 12-month-old infants’ expressive vocabulary, and that their parent-child shared reading experience appears to play a role in the formation of this predictive brain signal. Further mediation analysis showed that one third of the effect of early shared reading experience on expressive vocabulary was explained by infants’ predictive brain signals after controlling for maternal education. The final model explained 59.3% of the variance in infants’ expressive vocabulary abilities. These results suggest that aside from the direct influence on expressive vocabulary, richer shared reading experience also strengthens the predictive brain signals that in turn facilitates vocabulary growth in infancy.

The current finding on the importance of predictive neural responses on expressive vocabulary skills is in line with a previous fNIRS study [3]. Our findings are also consistent with theories about the role of predictive processing in shaping language processing mechanisms [e.g., 48] and computational modeling [e.g., 26]. Furthermore, the role of prediction in shaping educational outcomes emphasizes that preschoolers develop better language skills when given more opportunities to predict the forthcoming information during shared reading; for example, when parents used more strategic pauses to prompt children to predict upcoming words [49] or more frequently requested children to predict forthcoming events in the story [27, 28]. Taken together, the current and previous findings provide converging evidence for the hypothesis that prediction ability is part of the engine driving language development in early life.

Moreover, this is the first study identifying the effect of self-report early shared reading experience on the strength of infants’ predictive brain signals, and this effect is present as early as 12 months of age. These results are in line with past research showing that the benefit of shared reading experience can extend beyond language-related brain function to other domains such as executive function and social cognition [18, 50, 51]. As discussed previously, primary caregivers tend to spontaneously request their infants to predict future events based on the story during shared book reading [e.g., 27–29]. Shared book reading may particularly provide a setting encouraging ‘serve and return’ and ‘prediction making’ during parent-child interactions. Thus, when shared book reading is regularly engaged by parent-infant dyads at home, this consistent experience with prediction may enhance infants’ predictive abilities at the neural level.

Last and most importantly, the current study further provides evidence of a causal link between shared reading experience and expressive vocabulary that is mediated by the brain’s ability to make predictions in the first year of life. Theoretically, this novel finding reveals a neural mechanism by which early shared reading experience may influence the development of the brain’s predictive ability and in turn the development of child language. Namely, infants who have richer experience with shared reading activities exhibit greater brain predictive signals on the visual omission task, which in part explained the well-recognized link between shared reading experience and expressive vocabulary acquisition. Practically speaking, this novel finding implies the value of coaching parents to have dialogic interactions with their infants in a way that can provide increased opportunities for children to make predictions, such as asking predictive questions or using strategic pauses to prompt children to predict upcoming words.

Despite the statistical significance of the current study, two limitations should be noted. Firstly, given the demands of all neuroimaging methods (EEG, fNIRS, fMRI), subject attrition is typically as high as 50%. Although fNIRS is non-invasive and increases infants’ task compliance compared to other neuroimaging techniques that also measures cortical hemodynamic responses such as fMRI [52, 53], a substantial proportion of collected data were excluded from final analyses due to a range of reasons such as infants’ shorter attention span [54]. In the current study, the attrition rate is 43.9% (18 out of 41 infants’ collected data was excluded due to excessive movement or failure to watch a sufficient number of trials). Although this rate compares well with the average attrition rate of 34.2% reported in a recent meta-analysis with 272 experiments across 182 publications in infants aged under 24 months [54], future studies should attempt to modify the study design to reduce the attrition rate and increase generalizability. We would also note that the final sample consisted of relatively high SES families which may limit the generalizability of the current findings. Future studies can consider to include samples from more diverse SES backgrounds to see whether the current results can be replicated in samples from different SES backgrounds. And although maternal education was found to be the strongest determinant of child development among other SES indexes [44], it would be nice to add a wider range of SES indexes to obtain a comprehensive understanding of the role of SES in the link among shared reading experience, predictive brain signal and expressive vocabulary acquisition.

Secondly, shared reading experience was measured by the StimQ-READ subscale. While the StimQ-READ subscale has been successfully used in other studies investigating relations between shared reading experience and brain functions [17, 19], it had some limitations in the current study. One is that its reliance on parent report made it subjective to bias due to memory inaccuracy or social desirability (i.e., the tendency to report in a generally favor fashion). The other is that it did not measure specific interaction strategies that parents used when reading to their 12-month-old infants. As a consequence, it is not possible to make a strong inference between interactive strategies used by parents and how they are linked to the formation of predictive brain signal. Future studies should attempt to measure specific interaction strategies parents used by videotaping parent-child shared book reading activities.

In summary, the unique contribution of the current study is to reveal a novel viewpoint concerning the neural mechanisms mediating the association between shared reading exposure during infancy and expressive vocabulary at the age of 12 months. Namely, richer shared reading experience during infancy strengthens predictive signals in the infant brain and in turn facilitates expressive vocabulary. Our findings are not only theoretically significant but also practically relevant in terms of recommendations that parents should engage in regular reading to their children during infancy. Reading to children is a simple and powerful way to boost the brain predictive signals as well as language development even during infancy. Future studies should further investigate whether different interaction strategies used during shared reading may have differential impacts on infants’ predictive brain signals. Whether these results would differ when reading with mothers versus fathers also warrants further investigations. Such studies would help us understand how the developing brain responds to various styles of shared book reading and potentially provide neural evidence in support of and designing early interventions involving shared book reading.

## Supporting information

S1 File Supporting information for measures (S1 Appendix, S1 fNIRS task, S1 fNIRS data processing, S1 Identification of channels of interest). (DOCX)

S1 Data (SAV)
