# Supplementary material for: Predictive brain signals mediate association between shared reading and expressive vocabulary in infants
Source: PLoS One. 2022 Aug 3;17(8):e0272438. doi: 10.1371/journal.pone.0272438 (PMC9348734; doi:10.1371/journal.pone.0272438)
Supplement: S1 File — (DOCX) [file pone.0272438.s001.docx]

**Supporting information**

**S1 Appendix. Scoring system for the Infant version of StimQ-READ.**

| Q# | Questions | Scoring |
| --- | --- | --- |
| Q1 | number of books parent reads to child | “0” - no books  “1” - 1-9 books  “2” - 10-24 books  “3” - 25-49 books  “4” - 50 or more books |
| Q2 | number of board books parent reads to child | “0” - no books  “1” - 1-9 books  “2” - 10-24 books  “3” - 25-49 books  “4” - 50 or more books |
| Q3 | days per week parent reads to child | “0” - 0-1 days  “1” - 2-3 days  “2” - 4 days or more. |
| Q4 | whether parent reads book of nursery rhymes to child | “0” - “No” answer  “1” - “Yes” answer |
| Q5-Q10 | whether parent reads book about daily activities(Q5) /body parts(Q6) /simple shapes(Q7) /things around the house(Q8) /toys and favorite things(Q9) /animals (Q10) to child | For each question:  “0” - “No” answer  “1” - “Yes” answer |
| Q11 | whether parent reads book containing photographs of babies to child | “0” - “No” answer  “1” - “Yes” answer |
| Q12 | whether parent labels pictures while reading to child | “0” - “No” answer  “1” - “Yes” answer |

*Note.* The maximum score is 19. Q1-Q2 are questions related to reading frequency. Q4-Q11 are questions related to book content. Q12 is the question about usage of verbal labeling.

**S1 fNIRS task**

**Stimuli**

The stimuli are the same as those used in Emberson, Richards, and Aslin [1]. Auditory stimuli consist of two novel nonspeech sounds that are similar to a squeaky honk from a clown horn and an unusual rattle sound. Visual stimuli consist of a red cartoon smiley face that enter a white box from either the top or bottom of the box. Each of the two sounds are paired with one direction of movement, creating two pairs of audio-visual stimuli (A1V1, A2V2). The stimuli can be downloaded from the study of Emberson et al. [1].

**Stimulus presentation procedure**

The stimulus presentation procedure was adapted from Emberson et al. [1] and consisted of repeatedly paired auditory-visual events followed by rare auditory-only events (visual-omission trials). All trials started with the presentation of a gray screen with a darker gray box in the middle. The visual stimulus entered the box from either the top or the bottom, moving into the box to touch the opposite side of the box in 500ms, and then exiting the box in the same side that it entered from in another 500ms. The onset of the auditory stimulus was 750ms before the appearance of the visual stimulus from behind the box. Please see the study of Emberson et al. [1] for a sample of the screen.

**S1 fNIRS data processing**

First, invalid trials (i.e., non-looking trials) were discarded. Bad channels (via visual inspections) and channels showing very high or low optical readings were excluded from further analyses (using the function enPruneChannels). The raw intensity data were then converted to optical density (OD) changes. A motion detection filter was then applied to identify motion artifacts (using the function hmrMotionArtifactByChannel: tMotion = 1, tMask = 1, STDEVthresh = 15.5, AMPthresh = 0.4). These values were selected not only based on Di Lorenzo et al.’s suggestions, but also via visual inspection to ensure the majority of spike-like motion artifacts of this current data were identified. After motion detection, the combination of Spline (performed at first step; *p* = 0.99) and Wavelet (performed at second step; *iqr* = 0.8), an effective way of correcting motion artifacts in infant data recommended by Di Lorenzo et al., was applied for motion corrections. After correction, motion detection (using the same function hmrMotionArtifactByChannel described above) was applied again to identify the remaining uncorrected motion artifacts, then these uncorrected motion artifacts were further rejected. After this step, data were filtered using a band-pass filter between 0.01 Hz and 0.8 Hz. to reduce slow drifts and high-frequency noise. The OD data were then converted to concentration changes using the modified Beer-Lambert law [2].

S1 Fig. shows the time courses of oxy-Hb (red lines) and deoxy-Hb (blue lines) signals averaged over all infants during the two types of test trials (AV, AV-). The obtained data indicates that, during the two types of test trials, most of the channels in the occipital areas showed event-evoked increases in oxy-Hb during the time window between 0-3s after the auditory stimulus onset. Event-related hemodynamic responses for each measurement channel in the time window between 0-3s after the auditory stimulus onset during the test trials were individually obtained by averaging the changes in the oxy-Hb and deoxy-Hb signals respectively across data blocks for each infant and amplitudes of the responses to stimulation were evaluated at the group level.


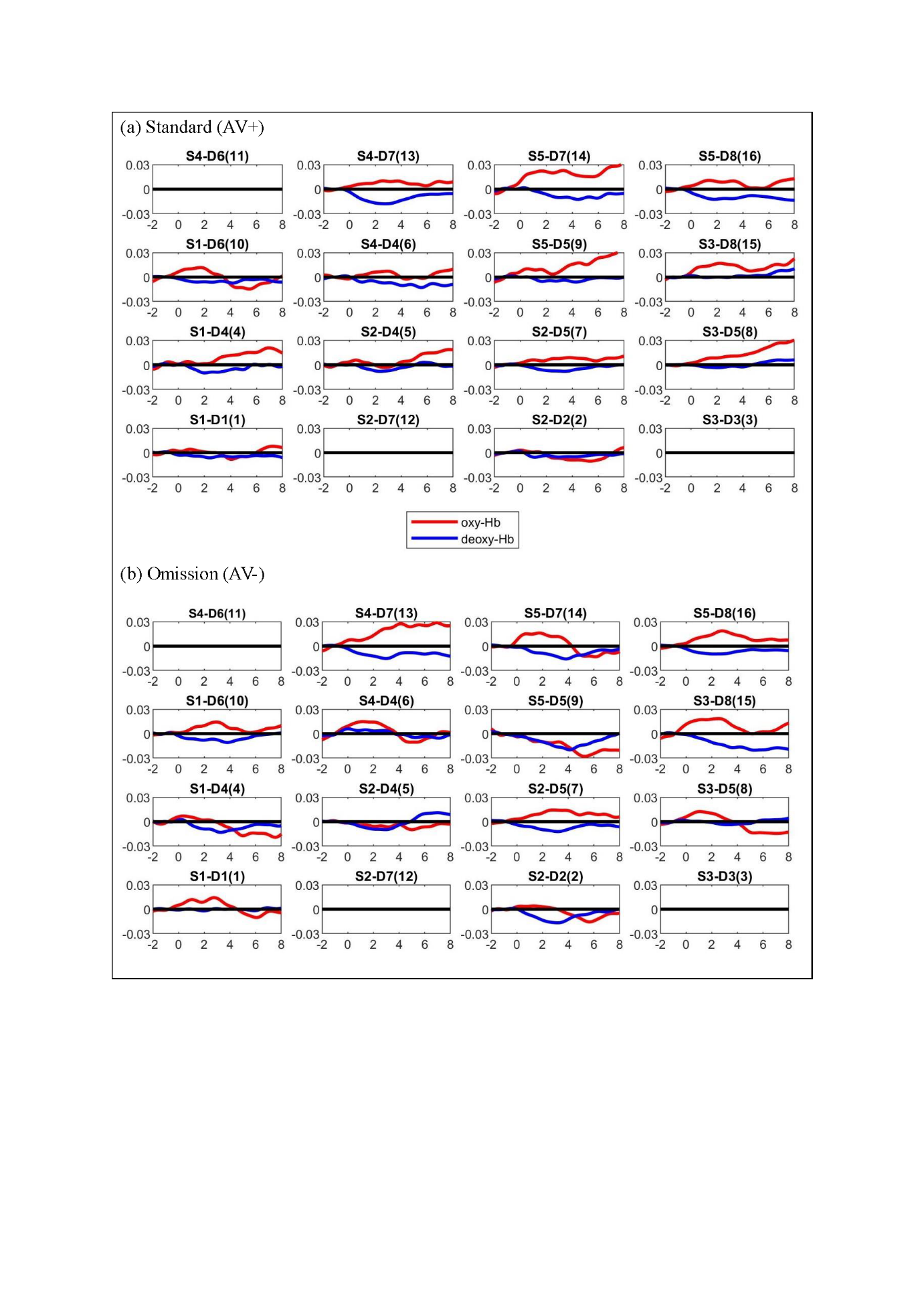


**S1 Fig.** Group-averaged time courses of changes in oxy-Hb (red lines) and deoxy-Hb (blue lines) (mM) signals in response to 1.75s stimuli presentation in (a) the standard audiovisual (AV) test trials and (b) the visual-omission (AV-) test trials. Numbers in brackets denote channel numbers. Data from channel 3, 11 and 12 were rejected in most of infants due to uncorrected motion artifacts, so no data were presented for these three channels. (color print)

**S1 Identification of channels of interest**

To characterize the predictive brain signals on AV- test trials, we first identified channels in the occipital area that showed significant activation to the AV test trials. One-sample t tests (two-tailed) of the oxy-Hb changes elicited by the AV test trials were compared to zero baseline for each channel covering the occipital brain areas. This way of identifying channels of interest has been used in previous studies [e.g., 3, 4]. Only oxy-Hb was used for the detection because in the infant literature it is typical that only oxy-Hb shows significant responses and deoxy-Hb fails to reach significance [5]. After multiple comparisons using the false discovery rate (FDR-HB) control (with the Benjamini-Hochberg critical value for a false discovery rate of 0.15) [6], three channels (14, 15, 16) were identified (*ps* < .05). Using the same statistical procedure, two channels (15, 16) were identified as the activated channels in the occipital area under the visual-omission condition (the AV- test trials). Channels showing significant changes during both types of single test trials were used for subsequent analyses (i.e., channel 15 and 16). According to fNIRS channel placement with reference to the 10-20 system, channel 15 and channel 16 approximately correspond to placement of PO4/P6/PO8 (see Fig. 2A). This right lateralized ROI is in line with a recent review suggesting that there is a generalized dominance of the right hemisphere for all functional conditions in the early stage of life (except linguistic stimuli) [7] and with theories stating that the right hemisphere develops earlier [8].

**S1 Reference**

1. Emberson LL, Richards JE, Aslin RN. Top-down modulation in the infant brain : Learning-induced expectations rapidly affect the sensory cortex at 6 months. Proceedings of the National Academy of Sciences of the United States of America. 2015;112(31):9585-90. doi: 10.1073/pnas.1510343112.

2. Cope M, Delpy DT. System for long-term measurement of cerebral blood and tissue oxygenation on newborn infants by near infra-red transillumination. Medical and Biological Engineering and Computing. 1988;26(3):289-94. doi: 10.1007/BF02447083.

3. Watanabe H, Homae F, Taga G. Activation and deactivation in response to visual stimulation in the occipital cortex of 6-month-old human infants. Developmental Psychobiology. 2012;54(1):1-15. doi: 10.1002/dev.20569.

4. Taga G, Watanabe H, Homae F. Spatiotemporal properties of cortical haemodynamic response to auditory stimuli in sleeping infants revealed by multi-channel near-infrared spectroscopy. Philosophical Transactions of the Royal Society A: Mathematical, Physical and Engineering Sciences. 2011;369(1955):4495-511. doi: 10.1098/rsta.2011.0238.

5. Issard C, Gervain J. Variability of the hemodynamic response in infants: Influence of experimental design and stimulus complexity. Developmental Cognitive Neuroscience. 2018;33:182-93. doi: 10.1016/j.dcn.2018.01.009.

6. Benjamini Y, Hochberg Y. Controlling the false discovery rate: A practical and powerful approach to multiple testing. Journal of the Royal Statistical Society Series B (Methodological). 1995;57(1):289-300. doi: 10.1111/j.2517-6161.1995.tb02031.x.

7. Bisiacchi P, Cainelli E. Structural and functional brain asymmetries in the early phases of life: a scoping review. Brain Structure and Function. 2021. doi: 10.1007/s00429-021-02256-1.

8. Geschwind N, Galaburda AM. Cerebral lateralization: Biological mechanisms, associations, and pathology: III. A Hypothesis and a Program for Research. Archives of Neurology. 1985;42(7):634-54. doi: 10.1001/archneur.1985.04060070024012.
